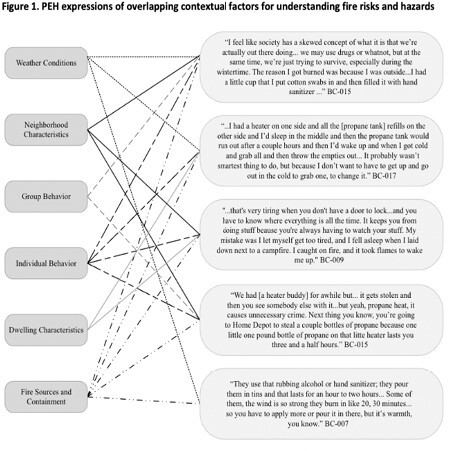# 577 Understanding Drivers of Fire Risk with People Experiencing Homelessness

**DOI:** 10.1093/jbcr/irae036.211

**Published:** 2024-04-17

**Authors:** Caitlin M Orton, Mark Gannon, Aldina Mesic, Mona Chambers, Tam N Pham, Barclay T Stewart

**Affiliations:** UW Medicine Regional Burn Center, Harborview Medical Center, Seattle, WA; The Night Ministry, Chicago, IL; University Of Washington, Hackettstown, NJ; UW Medicine - Harborview Burn Center, Seattle, WA; University of Washington, Harborview Burn Centre, Seattle, WA; University of Washington, Seattle, WA; UW Medicine Regional Burn Center, Harborview Medical Center, Seattle, WA; The Night Ministry, Chicago, IL; University Of Washington, Hackettstown, NJ; UW Medicine - Harborview Burn Center, Seattle, WA; University of Washington, Harborview Burn Centre, Seattle, WA; University of Washington, Seattle, WA; UW Medicine Regional Burn Center, Harborview Medical Center, Seattle, WA; The Night Ministry, Chicago, IL; University Of Washington, Hackettstown, NJ; UW Medicine - Harborview Burn Center, Seattle, WA; University of Washington, Harborview Burn Centre, Seattle, WA; University of Washington, Seattle, WA; UW Medicine Regional Burn Center, Harborview Medical Center, Seattle, WA; The Night Ministry, Chicago, IL; University Of Washington, Hackettstown, NJ; UW Medicine - Harborview Burn Center, Seattle, WA; University of Washington, Harborview Burn Centre, Seattle, WA; University of Washington, Seattle, WA; UW Medicine Regional Burn Center, Harborview Medical Center, Seattle, WA; The Night Ministry, Chicago, IL; University Of Washington, Hackettstown, NJ; UW Medicine - Harborview Burn Center, Seattle, WA; University of Washington, Harborview Burn Centre, Seattle, WA; University of Washington, Seattle, WA; UW Medicine Regional Burn Center, Harborview Medical Center, Seattle, WA; The Night Ministry, Chicago, IL; University Of Washington, Hackettstown, NJ; UW Medicine - Harborview Burn Center, Seattle, WA; University of Washington, Harborview Burn Centre, Seattle, WA; University of Washington, Seattle, WA

## Abstract

**Introduction:**

Burn injuries among people experiencing homelessness (PEH) are a major and escalating public health problem affecting a population facing issues of socioeconomic disparities, racism, comorbidities (e.g., disabilities, substance use disorders), and inequitable housing policies. Given the intensifying issue of burn injuries among PEH, it is crucial to Identify key drivers of fire risk in this already marginalized population to inform injury prevention and control initiatives.

**Methods:**

Key informant interviews (KII) with PEH who experienced burn injuries were conducted to identify fire risks and opportunities to mitigate hazards. Transcripts were coded using deductive and inductive strategies grounded in harm reduction theory. For the deductive component, an established conceptual model was applied to group factors into the following categories: weather conditions, neighborhood characteristics, group behavior, individual behavior, dwelling characteristics, and fire sources and containment. For the inductive component, emerging themes were identified and coded.

**Results:**

Participants from 13 KII had varied etiologies of their burn injuries (e.g., sleeping by fire, assault by fire, tent fire, car heater contact burn, propane tank explosion). The most common fire sources identified were propane, hand sanitizer, and other alcohol-based liquids. Unsafely contained open flames and improper propane tank storage were frequently expressed hazards. Participants described the interplay of environmental, social, and behavioral factors that need to be accounted for when addressing fire hazards (Figure 1). For example, participants highlighted the relationship between fire risks and acts of violence (e.g., theft of fuel sources, assault, forced relocation), as well as the multitude of considerations that impact fuel choice (e.g., accessibility, cost, smoke exposure, burn duration, flame control, weather conditions). Furthermore, participants described other specific opportunities for prevention within the intersectional context of the environments and risks they experience.

**Conclusions:**

A variety of fire hazards were identified and amenable to harm reduction (e.g., safe stove dissemination, education on safe storage, availability of warming shelters). To be effective, implementation of fire prevention and control strategies must be evaluated for their effectiveness in complex environments (e.g., exposure to severe weather, unsafe and potentially violent living conditions, substance use, social marginalization, and poor access to injury prevention media).

**Applicability of Research to Practice:**

Understanding environmental and socioeconomic risks and their interplay with fire hazards is foundational for collaborative injury prevention and control initiatives to be implemented by PEH, homelessness service providers, public health officials, and burn centers.